# Couples of significant others (COSO) in a joint effort to quit smoking

**DOI:** 10.1186/1617-9625-12-S1-A26

**Published:** 2014-06-06

**Authors:** Aikaterini Tsoutsa, Ioanna Nikoloutsou, Dimos Fotopoulos, Constantinos Glynos, Spyridon Zakynthinos, Paraskevi Katsaounou

**Affiliations:** 1 Pulmonary Department - ICU, Evangelismos Hospital, Athens, 10676, Greece

## Background

Motivational support is crucial for the success of smoking cessation. Significant others are a proven source of that support [1,2]. As far as we know social support has been used to achieve smoking cessation higher rates, but only as support and not as a concurrent attempt of a couple to quit smoking. We investigated whether the inclusion of couples of significant others in a joint effort to quit smoking in smoking cessation groups formed by a population based sample of participants would increase their succession rate compared to the participants that receive the same treatment alone.

## Materials and methods

This was a randomized population-based intervention study at the smoking cessation clinic of Evaggelismos hospital. We monitored for people that are related in the initial screening stage. Couples included life partners, family members or very close friends. Smokers were in all motivational stages. All participants underwent the same intervention with motivational and behavioural components in the smoking cessation groups and received medical consultation and pharmacotherapy (Varenicline). We compared so far the smoking cessation rates of 25 "couples" and 50 randomized smokers that followed our smoking cessation program.

## Results

We found that participants that joint the COSO quit smoking in a higher rate (58%) than of smokers (38%). Within the dyad the person more motivated to quit smoking was usually the first to quit. Among couples that quit smoking, men were more successful (63%) than women (49%).

## Conclusions

We conclude that higher smoking cessation rates were obtained in COSO joining our smoking cessation program.
